# Altered DNA Binding and Amplification of Human Breast Cancer Suppressor Gene *BRCA1* Induced by a Novel Antitumor Compound, [Ru(η^6^-*p*-phenylethacrynate)Cl_2_(pta)]

**DOI:** 10.3390/ijms131013183

**Published:** 2012-10-15

**Authors:** Korawan Chakree, Chitchamai Ovatlarnporn, Paul J. Dyson, Adisorn Ratanaphan

**Affiliations:** 1 Department of Pharmaceutical Chemistry, Faculty of Pharmaceutical Sciences, Prince of Songkla University, Hat-Yai, Songkhla 90112, Thailand; E-Mails: kchakree@hotmail.co.th (K.C.); lchitcha@pharmacy.psu.ac.th (C.O.); 2 Institute of Chemical Sciences and Engineering, Swiss Federal Institute of Technology Lausanne (EPFL), CH-1015 Lausanne, Switzerland; E-Mail: paul.dyson@epfl.ch

**Keywords:** *BRCA1*, DNA adducts, DNA amplification, ethaRAPTA, tumor suppressor gene

## Abstract

The ruthenium-based complex [Ru(η^6^-*p*-phenylethacrynate)Cl_2_(pta)] (pta = 1,3,5-triaza-7-phosphatricyclo-[3.3.1.1]decane), termed ethaRAPTA, is an interesting antitumor compound. The elucidation of the molecular mechanism of drug activity is central to the drug development program. To this end, we have characterized the ethaRAPTA interaction with DNA, including probing the sequence specific modified DNA structural stability and DNA amplification using the breast cancer suppressor gene 1 (*BRCA1*) of human breast and colon adenocarcinoma cell lines as models. The preference of ethaRAPTA base binding is in the order A > G > T > C. Once modified, the ethaRAPTA-induced *BRCA1* structure has higher thermal stability than the modified equivalents of its related compound, RAPTA-C. EthaRAPTA exhibits a higher efficiency than RAPTA-C in inhibiting *BRCA1* amplification. With respect to both compounds, the inhibition of *BRCA1* amplification is more effective in an isolated system than in cell lines. These data provide evidence that will help to understand the process of elucidating the pathways involved in the response induced by ethaRAPTA.

## 1. Introduction

Since the introduction of cisplatin [*cis-*dichlorodiammineplatinum(II)], and its analog carboplatin [*cis*-diammine-(1,1-cyclobutanedicarboxylato) platinum(II)] into clinical practice [1–4] there has been much interest in the development of anticancer drugs. Both platinum complexes have a similar spectrum of antitumor activity against human testicular, ovarian, urinary bladder and head and neck cancers [3] and the mechanism of cytotoxicity is mediated via covalent DNA binding between a platinum atom and the N-7 atom of guanine and guanine or adenine. The majority of platinum-DNA adducts are intrastrand crosslinks in the order Pt-GG (65%), Pt-AG (25%) to Pt-GNG (6%) [5,6]. Minor adducts include interstrand crosslink and the monofunctional adducts. All adducts interfere with DNA replication, transcription and translation, and finally leads to the apoptosis pathway and cancer cell death.

Unfortunately, the platinum-based anticancer complexes have some side effects such as nephrotoxicity, myelotoxicity, ototoxicity, peripheral neuropathy, nausea and vomiting, myelosuppression and thrombocytopenia [3,7]. Furthermore, their clinical utility is limited, and many tumor cells are resistant to platinum drugs [8,9]. Where platinum drugs are ineffective, the mortality from cancer is significantly higher than when they are effective. For these reasons, new transition metal-based compounds are being designed that overcome the platinum complex limitations. Ruthenium complexes have properties particularly well suited for medicinal applications [10], including relevant ligand exchange kinetics, redox potentials and the ability to mimic iron in binding to certain biological molecules [11]. Currently, several generations of ruthenium complexes have been synthesized and investigated for their anticancer properties [12]. These ruthenium complexes have a different mode of action than the platinum-based drugs and could therefore widen the range of cancers that can be treated.

**Figure 1:**
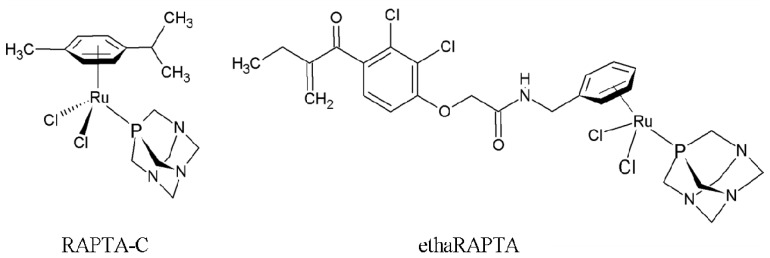
Structures of the RAPTA complexes.

The ruthenium complexes imidazolium *trans*-[tetrachlorido(1*H*-imidazole)(*S*-dimethyl sulfoxide) ruthenate(III)] (NAMI-A) and indazolium *trans*-[tetrachloridobis(1*H*-indazole)ruthenate(III)] (KP1019) are among the most promising anticancer candidates [12]. Both NAMI-A and KP1019 have successfully completed phase 1 clinical trials and NAMI-A entered phase 2 clinical trials in 2008 (in combination with a cytotoxic drug), and KP1019 is also undergoing further clinical trials [13]. The RAPTA compounds contain a ruthenium(II)-arene unit with a PTA ligand [14]. These complexes have been shown to interact with DNA in a pH-dependent manner [15,16] and offer cancer cell specific targeting. In the case of the cytotoxicity of the RAPTA complexes on TS/A mouse adenocarcinoma cells and on HBL-100 cells (normal human breast epithelial cells), the RAPTA complexes are selective towards the TS/A cells whereas cytotoxicity on the HBL-100 cells was not observed [10]. The selective cytotoxicity of these RAPTA complexes toward the cancer cells could potentially lead to a drug with lower side effects compared to other metal-based anticancer drugs [10]. Although the rate of reaction with DNA is significantly lower than that of cisplatin, several RAPTA complexes display excellent *in vivo* activity [10,17]. The prototype compound, [Ru(η^6^-*p*-cymene)Cl_2_(PTA)], termed RAPTA-C (Figure 1), remains the best characterized complex in the series and the molecular mechanism has been shown to involve mitochondrial-induced apoptosis [18]. However, the rate of reaction of RAPTA-C with DNA is significantly lower than those of cisplatin or carboplatin. Recently, organometallic ruthenium complexes containing ethacrynic acid (EA) ligands have been rationally developed as drugs which selectively bind to glutathione *S*-transferase (GST) and inhibit GST activity [19]. With the validation of ethacraplatin to overcome glutathione *S*-transferase (GST)-mediated drug resistance, a new RAPTA complex with EA tethered to the arene ring [(ethacrynic-η^6^-benzylamide)RuCl_2_(pta) or ethaRAPTA] (Figure 1), was therefore developed, and investigated for GST-inhibitory activity and its effect on the proliferation of cancer cells. EthaRAPTA was found to inhibit cancer cell growth (A549, HT29, MCF-7, A2780, and A2780cisR cell lines) [19]. However, the molecular mechanisms underlying the ethaRAPTA-induced response remain to be elucidated.

*BRCA1* is an important tumor suppressor gene in humans. Its translated product, the 220 kDa BRCA1 protein, has been shown to play an important role in genomic integrity maintenance such as DNA repair, cell-cycle checkpoint control, transcriptional regulation and protein ubiquitination [20–22]. Therefore, approaching such a gene as a potentially molecular target for the antitumor ruthenium(II)-arene (RAPTA) compounds might be of interest in cancer therapy. Recently, the interaction of two RAPTA compounds, RAPTA-C and carboRAPTA-C, with the specified DNA sequence of the human breast cancer suppressor gene *BRCA1* has been studied [23]. The ruthenation of DNA by RAPTA-C was also very similar to the platination value observed for carboplatin. Both RAPTA-C and carboRAPTA-C formed different ruthenium-DNA adducts with predominantly monofunctional adducts at A and C and, to a lesser extent, at G, which contrasts with the behavior of cisplatin [24]. For ethaRAPTA, the Ru-modified *BRCA1* may lose its functions in cancerous cells that ultimately result in cancer cell death. In the present study, we investigate the interactions of ethaRAPTA with the specified DNA sequence of the human *BRCA1* gene in cells and a cell-free system.

## 2. Results and Discussion

### 2.1. EthaRAPTA-Mediated Conformational Changes of the Cell-Free BRCA1 Fragment

**Figure 2:**
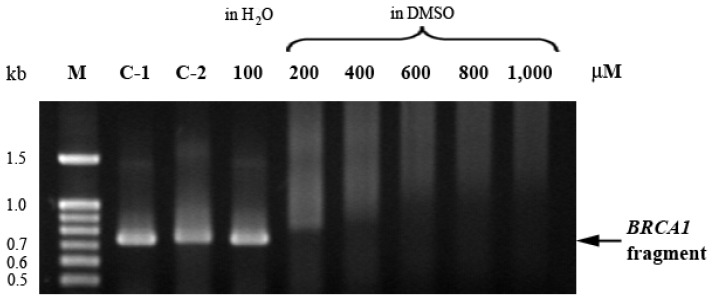
Electrophoretic mobility of ethaRAPTA-treated *BRCA1* fragment. The 696 bp *BRCA1* fragment (3 μg) was incubated with various concentrations of ethaRAPTA (100–1000 μM) at 37 °C for 24 h in the dark. Ruthenated DNA was electrophoresed on 1% agarose gel. The gel was stained with ethidium bromide and visualized under UV light. M = 100 bp DNA ladder, C-1 = untreated DNA (DNA solution in sterile double distilled water), C-2 = untreated DNA (DNA solution in DMSO).

The 3′-terminal region of the human *BRCA1* gene covering exon 16–24 (nucleotide 4897–5592) was used as a model for the ethaRAPTA-mediated retardation of DNA. The electrophoretic mobility of ethaRAPTA-treated *BRCA1* fragment was reduced as the concentration of ethaRAPTA increased (Figure 2). Ruthenation caused a progressive increase in the frequency of DNA lesions. It is interesting to note that at a ruthenium concentration of 200 μM, the band disappeared due to aggregation of the ethaRAPTA-*BRCA1* adducts and thus inhibited the intercalation of ethidium bromide into the DNA molecules at higher levels of ruthenation.

**Figure 3:**
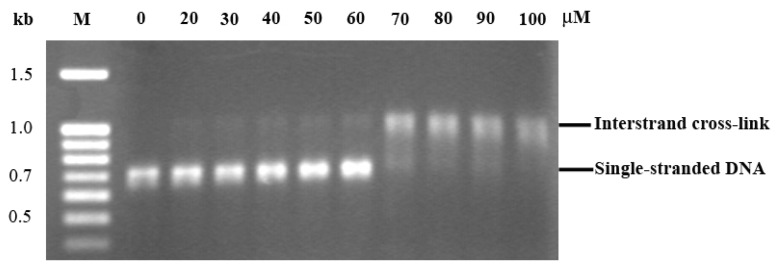
Interstrand crosslinks between ethaRAPTA and the *BRCA1* fragment. The 696-bp *BRCA1* fragment (3 μg) was incubated with various concentrations of ethaRAPTA (20–100 μM) at 37 °C for 24 h in the dark. Ruthenated DNA was electrophoresed on 1% alkaline agarose gel. The gel was neutralized by neutralizing solution, stained with ethidium bromide and visualized under UV light.

Gel electrophoresis was used to study the effect of ethaRAPTA on DNA interstrand crosslinks. Under alkaline denaturation, double-stranded DNA is disrupted to a single strand. Both strands migrate similarly as the rate is dependent on the size and not the sequence of DNA. The mobility of the DNA strands began to change at the ruthenium(II) complex concentration of 50 μM and this change was complete at the concentration of 80 μM (Figure 3). At ethaRAPTA concentration of 50 μM, initiation of interstrand crosslinks was formed. The intensity of interstrand crosslinks increased as ethaRAPTA concentration increased. The complete interstrand crosslinks were formed at ethaRAPTA concentration of 80 μM. At a ruthenium concentration of 70 μM or above, the band intensity was reduced due to aggregation of the ethaRAPTA-*BRCA1* adducts and thus inhibited the intercalation of ethidium bromide into the DNA molecules at higher levels of ruthenation.

### 2.2. The Preference of ethaRAPTA Base Binding is in the Order A > G > T > C

**Figure 4:**
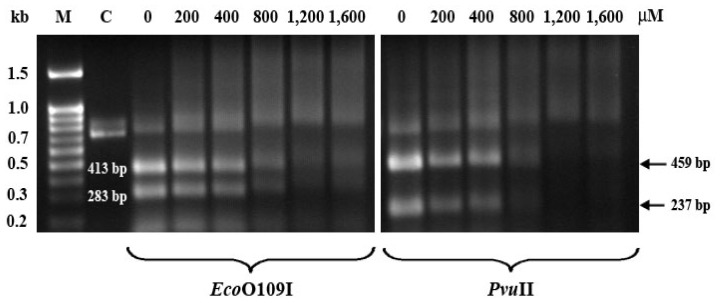
Restriction analysis for ruthenation site of the 696-bp *BRCA1* fragment. The 696-bp *BRCA1* fragment (3 μg) was incubated with various concentrations of RAPTA-EA1 (200–1600 μM) at 37 °C for 24 h in the dark. The ethaRAPTA-treated DNA was precipitated, redissolved in steriled double distilled water and further incubated with either *Eco*O109I or *Pvu*II at 37 °C for 5 and 6 h, respectively. Restriction products were electrophoresed on 1% agarose gel. The gel was stained with ethidium bromide and visualized under UV light. M = 100 bp DNA ladder, C = untreated and uncut DNA.

Preferential sites for ruthenation on the 696-bp fragment of the 3′-terminal region of *BRCA1* may be deduced from restriction analysis using specific enzymes (*Eco*O109I and P*vu*II) that recognize specific sequences on the tested gene. The *Eco*O109I cleaved the gene fragment into two fragments of 283-bp and 413-bp and the digestion of *Pvu*II produced two digested fragments of 237-bp and 459-bp which can be detected by electrophoresis on an agarose gel. Production of the digested fragments from ethaRAPTA-treated DNA in the presence of both *Eco*O109I (dGpG) and *Pvu*II (dGpC) was inhibited at similar concentrations, in a dose-response behavior (Figure 4). At 800 μM or above, the ethaRAPTA-treated *BRCA1* fragment became resistant to both restriction enzymes. Both enzymes showed a similar level of inhibition and with no specificity between the two restriction sites. However, these two enzymes were about twice less effective in restriction cleavage when compared with its prototype RAPTA-C-treated *BRCA1* fragment [23], indicating that the large bulky group of the ruthenium center may hinder accessibility by the enzymes to their restriction sites on the DNA molecules. In addition, the band intensity was reduced due to aggregation of the ethaRAPTA-*BRCA1* adducts and thus inhibited the intercalation of ethidium bromide into the DNA molecules at higher levels of ruthenation.

**Figure 5:**
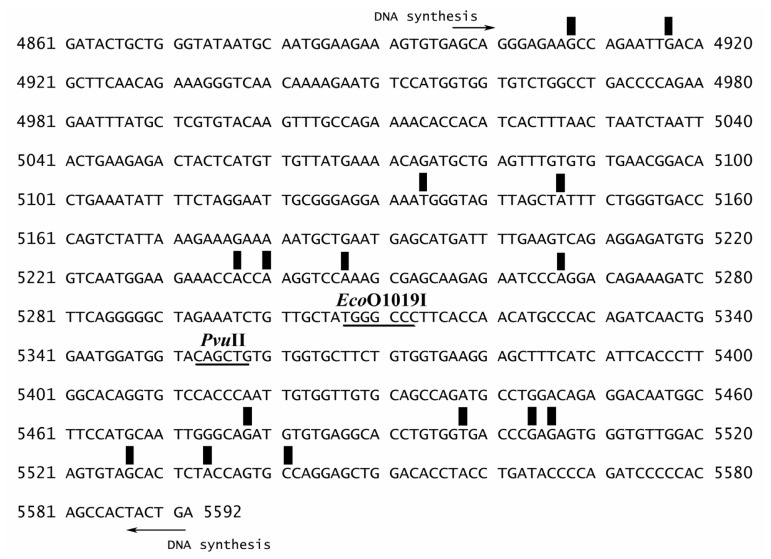
Schematic diagram showing base/sequence of the *BRCA1* fragment used to monitor the inhibition of DNA synthesis on the template modified by ethaRAPTA. The arrow indicates the start site and the direction of DNA synthesis. The bars represent possible monofunctional crosslinks. TGGGCCC is a recognition sequence of *Eco*O109I and CAGCTG is a recognition sequence of *Pvu*II. Numbers represent the nucleotide sequence of cDNA of *BRCA1* gene (exon 16–24).

The sequence specificity of ethaRAPTA-*BRCA1* adducts was determined. Sequence analysis showed that ethaRAPTA was preferentially attacked by the *BRCA1* gene in the order A > G > T > C. A possible interstrand crosslinking between the ruthenium atom and the base/sequence of the *BRCA1* fragment is shown in Figure 5. However, no ethaRAPTA-*BRCA1* adducts were observed at the *Eco*O109I or *Pvu*II cleavage sites suggesting that the ruthenation with the accessibility or function of these endonucleases. The observed ruthenation sites in this study agree very well with NMR, MS and gel electrophoresis studies, suggesting that RAPTA, NAMI-A and KP1019 exhibit similar reactivity towards adenine and guanine [13,14]. In addition, it was found that adduct formation of the *BRCA1* fragment induced by RAPTA-C or carboRAPTA-C occurred most frequently at A, C, and G, in that order [23]. These crosslinks can be expected based on their different properties of the ruthenium complexes [25].

### 2.3. EthaRAPTA can also Bind to the *BRCA1* Fragment through Intercalation

**Figure 6:**
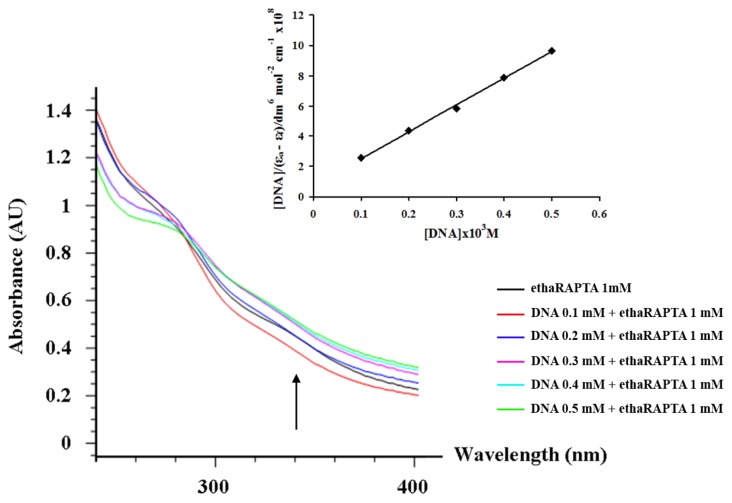
Absorption spectra of ethaRAPTA complex ([Ru] = 1 mM) in the presence of increasing concentrations of the 696-bp *BRCA1* fragment. The arrow indicated the changes in absorbance upon increasing the DNA concentration. Inset: plot of [DNA]/(*ɛ*_a_ − *ɛ*_f_) *vs.* [DNA].

The absorption spectra of ethaRAPTA, in the absence and the presence of the *BRCA1* fragment (at a constant concentration of complexes; 1000 μM), is shown in Figure 6. The absorption intensities of ethaRAPTA were shown to successively increase and exhibited hyperchromism. This result is similar to that reported for the interaction of other types of ruthenium(II) complexes [26–29]. The intrinsic binding constant (*K*_b_) of the complex with the *BRCA1* fragment was determined as 2.34 × 10^6^ M^−1^ from the changes in absorbance at 340 nm using the Equation 1 (see Experimental Section). As a result, ethaRAPTA can also bind to the *BRCA1* fragment through intercalation [30].

### 2.4. Altered Thermal Stability of EthaRAPTA Adducts

**Figure 7:**
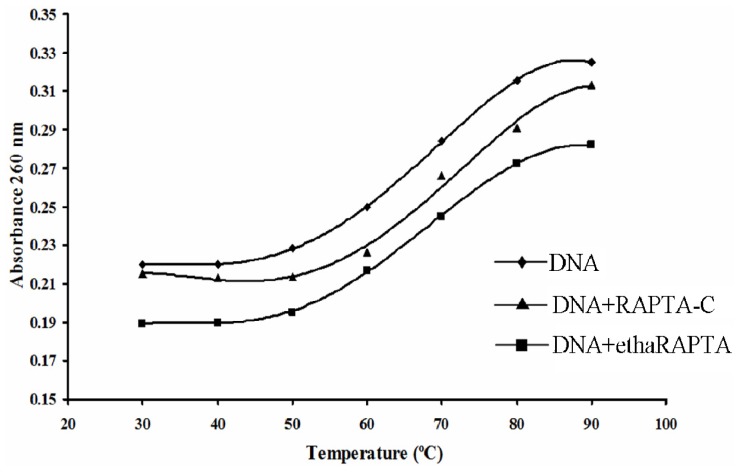
Thermal denaturation of the 696-bp *BRCA1* fragment in the absence (◆) or in the presence of RAPTA-C (▲) and ethaRAPTA (■). The 696-bp *BRCA1* fragment (3 μg) was incubated with either ethaRAPTA or RAPTA-C at 37 °C for 24 h in the dark. Thermal denaturation was obtained by measuring the absorbance at the wavelength of 260 nm with the temperature scanning. The melting temperature (*T*_m_) was taken as a mid-point of the melting curves.

The DNA melting technique is indeed a very popular, sensitive and easy tool to detect even slight DNA conformational changes induced by metal complexes and other ligands [31]. As temperature is increased, the double-stranded DNA gradually dissociates to a single strand, and generates a hypochromic effect on the absorption spectra of DNA bases (λ_max_ = 260 nm). The melting temperature (*T*_m_), which is defined as the temperature where half of the total base pairs are dissociated, is determined from the thermal denaturation curves of DNA. Difference in melting temperature of DNA treated and non-treated by metal complexes can give an insight into the nature of the interaction of the metal complexes with DNA. The Δ*T*_m_ values are small and positive for covalent adducts of monoaqua complexes and are large and positive for adducts of diaqua complexes [32]. The positive values of Δ*T*_m_ imply that the covalent adduct is more difficult to melt than the unbound DNA. The DNA melting curves for the *BRCA1* fragment in the absence or in the presence of the ruthenium(II) complexes were shown in Figure 7. When compared with the untreated DNA, the melting temperature increased upon treatment with either RAPTA-C or ethaRAPTA, respectively. An increase in DNA melting temperature may reflect an increased stability of the helical DNA structure after binding to these RAPTA complexes.

### 2.5. EthaRAPTA Exhibited a Higher Efficiency than RAPTA-C in Inhibiting *BRCA1* Amplification

**Figure 8:**
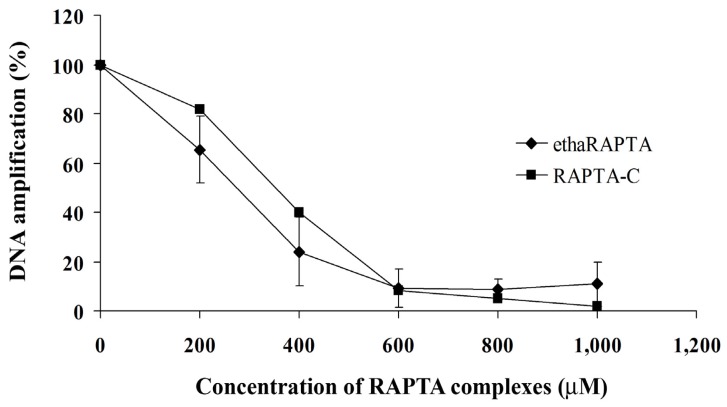
Amplification products of DNA treated by ethaRAPTA and RAPTA-C were quantified, and the amount of DNA amplification (%) was plotted as a function of concentration.

In order to detect the degree of DNA damage, the quantitative PCR (QPCR) method was used to monitor the progress of the *Taq* DNA polymerase in a PCR that utilized the DNA adducts as templates. Previous reports have shown that the QPCR assay can be used to study cellular DNA damage in the specific genes after exposure to DNA damaging agents, such as cisplatin [33], carboplatin [33], nitrogen mustards [34,35], UV irradiation [36], chlorambucil [37], alkylbenzylguanine [38], and 2-chloro-2-deoxyadenosine [39]. QPCR is a reliable method, equivalently sensitive to the ICP-MS method to analyze the incorporation of platinum-based drugs into the gene of interest [33]. The PCR was carried out under conditions that allowed for exponential amplification such that DNA damage will significantly reduce the amount of the amplified product. A QPCR study for ruthenium complexes has been previously reported and indicated that the ruthenium complexes reduced the amount of amplified DNA compared to the control untreated DNA [23]. As shown in Figure 8, the plots showed that DNA amplification decreased as the concentration of ethaRAPTA increased. DNA amplification was reduced by half at an approximate concentration of 300 μM for both ruthenium complexes. However, ethaRAPTA was only slightly more effective than RAPTA-C at blocking DNA replication.

**Figure 9:**
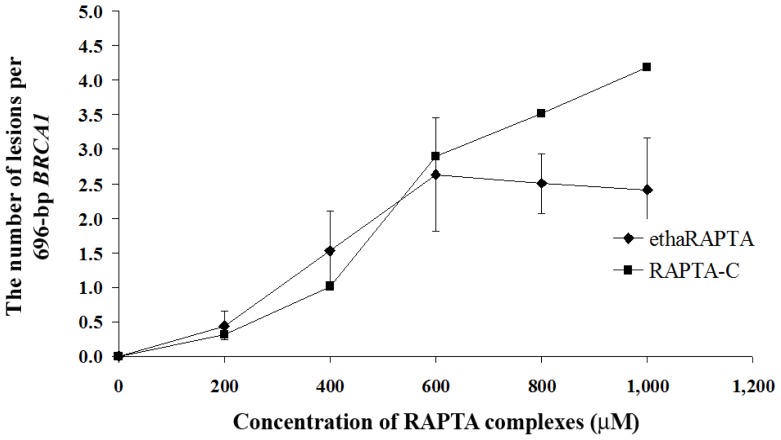
The number of lesions per the *BRCA1* fragment induced by ethaRAPTA and RAPTA-C. Absorbance units were applied to a Poisson equation and plotted as a function of the concentration.

Induction of lesions with the 696-bp *BRCA1* fragment can be quantitated by assuming a random (Poisson) distribution of damage [33]. The amounts of lesions per the *BRCA1* fragment are calculated using the Poisson equation, Equation 2, as described in the Experimental Section. A relationship between the concentration of ethaRAPTA and ruthenated DNA is illustrated in Figure 9 and is used to estimate the amounts of DNA lesions in the *BRCA1* fragment. The results showed approximately one lesion per the *BRCA1* fragment at 50% inhibition of DNA amplification.

**Figure 10:**
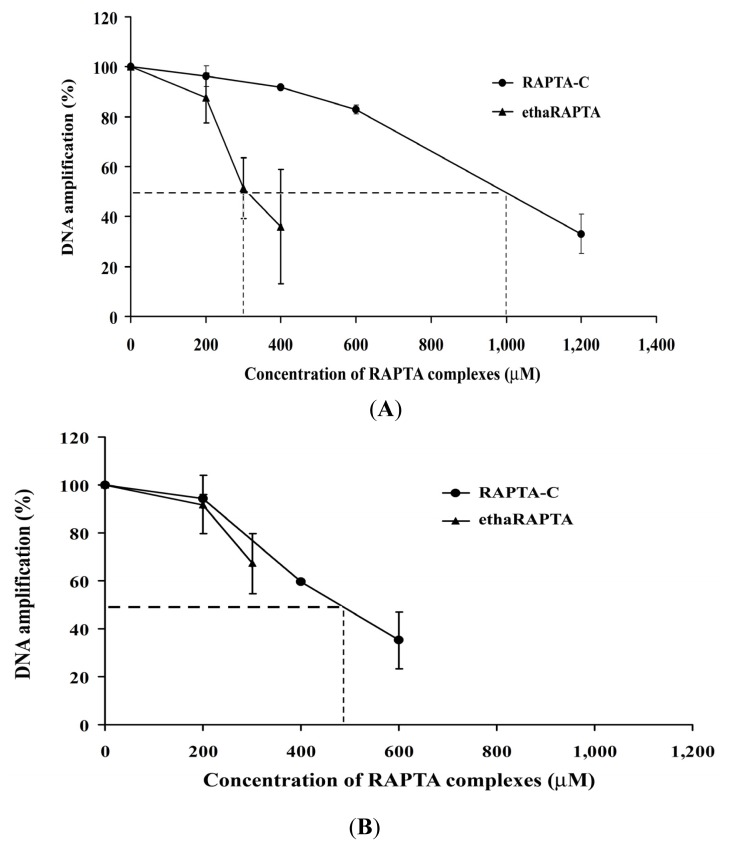
Cellular *BRCA1* damage in MCF-7 (**A**) and HT-29 (**B**) cells. The cells were incubated with various concentrations of ethaRAPTA (200–400 μM) and RAPTA-C (200–1200 μM), respectively, at 37 °C for 48 h in 5% CO_2_. Genomic DNA of the ruthenium-treated or untreated cells was isolated, and the 3426-bp fragment of the *BRCA1* exon 11 of the cells was then amplified by PCR, electrophoresed on 1% agarose gel, stained with ethidium bromide and then visualized under UV light. The amplification products were quantified using a Bio-Rad Molecular Imager, and the amount of DNA amplification (%) was plotted as a function of the concentration.

**Figure 11:**
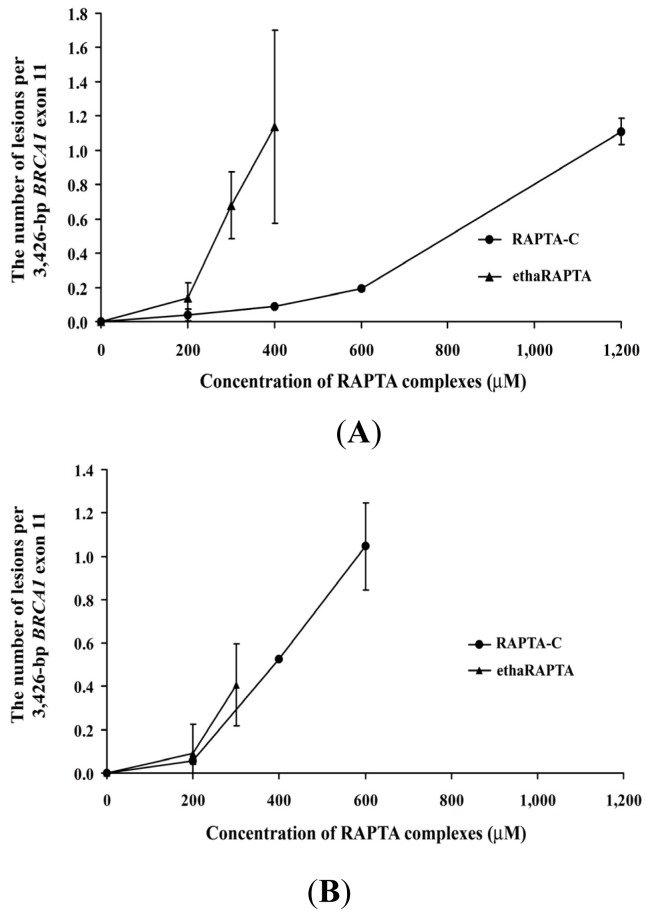
The number of lesions per the 3426-bp fragment of the *BRCA1* exon 11 in MCF-7 (**A**) and HT-29 (**B**) cells calculated by the Poisson equation.

For cellular DNA damage, the MCF-7 or HT-29 cell lines were incubated with either ethaRAPTA or RAPTA-C before genomic DNA was extracted and analyzed. As shown in Figure 10A,B, two RAPTA complexes significantly reduced *BRCA1* amplification of both the MCF-7 and HT-29 cells. The total amount of amplified PCR product was inversely proportional to the amount of DNA adducts within the specified *BRCA1* fragment (Figure 11A,B).

EthaRAPTA blocked 50% DNA amplification of the *BRCA1* exon 11 of MCF-7 cells at a concentration of 300 μM and that of HT-29 cells above 300 μM. Similarly, RAPTA-C exerted its inhibitory effect on DNA amplification at a concentration of 1000 μM and 500 μM for MCF-7 and HT-29 cells, respectively. However, an approximate 6- to 15-fold higher concentration of ethaRAPTA was required to achieve the 50% inhibition of DNA amplification in both cell lines. In addition, the ethacrynic acid alone did not interfere with DNA amplification (data not shown).

The inhibition concentration of 50% cancer cell growth, as shown in Table 1, by ethaRAPTA was lower than that by RAPTA-C both in MCF-7 and HT-29 cell lines, indicating a higher efficiency of ethaRAPTA over RAPTA-C in inhibiting these cancer cell growths. The increased efficiency may be a function of the ligand substitution on an arene group of these RAPTA complexes and nature of the cells [19,40,41]. In ethaRAPTA, ethacrynate is hydrophobic [42], making the complex more hydrophobic than that of the RAPTA-C. Furthermore, ethacrynic acid has a unique ability to inhibit glutathione *S*-transferase [43], particularly with GST P1-1 [19,30,41,44,45] offering increased potency via protection against detoxification. Crystallographic studies showed ethaRAPTA coordinated with both Cys101 residues from the homodimer of GST P1-1 via a loss of the chloride ligands. The ethacrynate ligand inserted into the hydrophobic substrate binding site (H-site), which is part of the active site [46,47], produced an interaction that suggests the disruption of the c-Jun *N*-terminal kinase (JNK) inhibition elicited by GST P1-1 enzyme [48].

It is observed that the degree of inhibition of DNA amplification by these two RAPTA complexes was lower in cancerous cells than in the isolated systems, possibly because of the interaction of the metal complexes with participating proteins [45] or the structure of the histone-bound DNA offering some protection [49]. It was also noted that in some incubations (ethaRAPTA above 300 μM and RAPTA-C above 600 μM, as shown in Figure 10B), the RAPTA complexes precipitated in the growth medium of the cell culture. In addition, ethaRAPTA has been shown to interact with proteins in the cell culture medium and such interactions are favored over DNA binding [30]. It is likely that ethaRAPTA interacts with numerous targets [17,30,45,50–56]. In addition, ethaRAPTA has been demonstrated to induce apoptosis in the cisplatin-resistant MCF-7 breast cancer cell line. This suggested that ethaRAPTA triggers multiple pathways toward apoptosis, including those involving endonuclease G, caspases, and c-Jun *N*-terminal kinase [46]. These data provided insights into the mode of action of ethaRAPTA to participating proteins as opposed to DNA [49]. Therefore, it could be possible that DNA is not the primary target for ethaRAPTA, based on our data of the inhibition of DNA amplification.

## 3. Experimental Section

### 3.1. Materials

EthaRAPTA and RAPTA-C were prepared using literature protocols [15,41]. *Taq* DNA polymerase, *Pvu*II, *Eco*O190I, dNTPs and Tris-HCl were from New England Biolabs. The nucleotide sequences of the forward and reverse primers were obtained from Invitrogen; RT-PCR: forward primer, 5′-AGCAGGGAGAAGCCAGAATTG-3′ and reverse primer, 5′-TCAGTAGTGGCTGTGG GGGAT-3′; 696-bp *BRCA1*: forward primer, 5′-ATAAAATCGACAGGGATCCTTAGCAGGGAG AAGCCAGAATTG-3′ and reverse primer, 5′-ACTTTGTGTTCATTTTCTAGATCAGTAGTGGCTG TGGGGGAT-3′; 3426-bp *BRCA1* exon 11: forward primer, 5′-GCCAGTTGGTTGATTTCCACC-3′ and reverse primer, 5′-GTAAAATGTGCTCCCCAAAAG-3′. All other reagents were of the highest purity grade.

### 3.2. Preparation of the 696-bp *BRCA1* Fragment (Exon 16–24, Nucleotide 4897–5592)

RNA (mRNA) was extracted from white blood cells using an mRNA isolation kit and biotinylated oligo-dT (*Qiagen*). The purified mRNA was used for complementary DNA (cDNA) synthesis and the amplification of the 696-bp *BRCA1* fragment (nucleotide 4897–5592) was performed with the *Qiagen OneStep RT-PCR Kit* (*Qiagen*). The RT-PCR mixture was prepared in a 1.5-mL microcentrifuge tube with a final volume of 50 mL, containing a 5 mL reaction buffer, 400 μM of each dNTP, 0.6 μM of forward primer, 0.6 μM of reverse primer, *OneStep* RT-PCR enzyme mix (*Qiagen*), and RNase-free H_2_O. The template RNA was finally added in order to initiate the PCR reaction using two-step thermal cycling. The first step comprised of one cycle at 48 °C for 45 min, allowing for the synthesis of the first strand cDNA by the action of reverse transcriptase. The reverse transcriptase was inactivated at 94 °C for 2 min. The second step included 40 cycles of denaturation at 94 °C for 30 s, annealing at 60 °C for 1 min, and extension at 68 °C for 2 min. The extension of the final cycle was expanded to 7 min. The amplified PCR product was subjected to gel electrophoresis in agarose at 80 V for 60 min. The gel was stained with ethidium bromide (EB), visualized under ultraviolet light (UV), and the DNA extracted with a gel extraction kit (*Qiagen*). The sequence of the purified 696-bp *BRCA1* fragment was subsequently verified using an automated DNA sequencer (ABI PRISM™ 377 DNA Sequencer) with a PRISM™ Ready Reaction Dye Deoxy Terminator Cycle Sequencing Kit (Applied Biosystem, Foster City, CA, USA).

To avoid the interaction between ruthenium atoms and salts in buffers, the purified 696-bp *BRCA1* fragment (3 μg) used in the following experiments was dissolved in double-distilled water and then incubated with various concentrations of ethaRAPTA at 37 °C for 24 h in the dark. Ruthenated DNA was further characterized for adduct formation and *in vitro* inhibition of DNA amplification.

### 3.3. Sequence Preference for EthaRAPTA Binding to the *BRCA1* Fragment

The ethaRAPTA-treated *BRCA1* fragment control (non-ruthenated) was ethanol precipitated, resuspended in double-distilled water and the concentration of DNA was determined spectrophotometrically at 260 nm. Ruthenium-treated DNA (200 ng) was mixed with *BigDye* terminator in a PCR tube (20 μL) containing 5 pmol of forward primer (5′-GGAATTCCATA TGAGCAGGGAGAAG-3′) or reverse primer (5′-ATTGGTTCTGCAGRCAGTAGTGGCT-3′), 200 μM of each dNTP, 1.5 mM MgCl_2_, 1 mM Tris-HCl (pH 8.3), and 1 unit of *Taq* DNA polymerase. The reactions were subjected to temperature cycling using a *Perkin-Elmer Model* 9600 cycle (Applied Biosystem, Foster City, CA, USA). The reactions used followed the protocols in the literature [23].

### 3.4. Absorption Titration

Absorption spectra were recorded on a Hewlett-Packard 8452A Diode Array Spectrophotometer using a cuvette of 10 mm path length. The absorption titration was performed with a solution of 1 mM ethaRAPTA in DMSO. The DNA solution of 4–17 μL (1 × 10^−4^–5 × 10^−4^ M) was added to the sample cell. The sample solution was mixed and allowed to equilibrate for 15 min before measurement. The data were then fitted to the following equation, Equation 1, to obtain the intrinsic binding constant, *K*_b_ [57].

(1) $$\left[\text{DNA}]/({ɛ}_{\text{a}}-{ɛ}_{\text{f}})=[\text{DNA}]/({ɛ}_{\text{b}}-{ɛ}_{\text{f}})+1/K_{\text{b}}({ɛ}_{\text{b}}-{ɛ}_{\text{f}}\right)$$

where ɛ_a_, ɛ_f_ and ɛ_b_ are the apparent, free and bound metal complex extinction coefficients, respectively. From a plot of [DNA]/(ɛ_a_ − ɛ_f_) *vs.* [DNA], a slope of 1/(ɛ_b_ − ɛ_f_) is obtained. *K*_b_ is the ratio of slope and the intercept.

### 3.5. Thermal Denaturation of RAPTA-BRCA1 Adducts

The 696-bp *BRCA1* fragment (3 μg) was incubated with 60 μM of ethaRAPTA or 30 μM RAPTA-C in 1000 μL of a reaction mixture at 37 °C for 24 h in the dark. Thermal denaturation profiles were obtained by measuring the absorbance at 260 nm for solutions of the 696-bp *BRCA1* fragment in the absence and presence of the RAPTA complexes with the temperature scanning of 20–100 °C. The melting temperature (*T*_m_) was taken as the mid-point of the melting curves.

### 3.6. Quantification of DNA Lesions Using QPCR

The quantitative PCR (QPCR) method was used to assess the polymerase inhibiting effect of DNA ruthenation. RAPTA-treated DNA was ethanol precipitated and centrifuged at 12,000*g* at 4 °C for 20 min. After drying, the DNA pellet was resuspended in 10 μL of double distilled water and the concentration of DNA was determined spectrophotometrically at 260 nm. The PCR mixture (50 μL) contained 100 ng of Ru-treated DNA, 0.5 μM of each forward and reverse primer, 200 μM of each dNTP, 2 mM MgCl_2_, and 1.5 units of *Taq* DNA polymerase. The PCR conditions were as follows: 3 min at 94 °C, 30 cycles of 45 s at 60 °C, 45 s at 72 °C, and a final extension for 10 min. The PCR products were separated by gel electrophoresis on 1% agarose at 80 V for 60 min. The gel was stained with ethidium bromide and visualized under UV light.

Product amplification was measured directly from the agarose gel using a Bio-Rad Molecular Imager with Image Quant Software Bio-Rad GS-700 Imaging Densitometer with the Molecular Dynamics program (version 1.0.2, Bio-Rad, Hercules, CA, USA, 1994). The amount of amplification was represented by the units of absorbance of the amplified products. The quantitative PCR assay was further employed to estimate the number of lesions per strand. Based on the assumption that the lesions were randomly distributed [33], the Poisson equation, Equation 2, was used to calculate the lesion frequency per strand.

(2) $$S=-\text{ln}(A_{\text{d}}/A)$$

Where *S* is the lesion frequency/strand, *A* is the absorbance unit produced from a given amount of non-damaged DNA template, and *A*_d_ is the absorbance unit produced from a given amount of damaged DNA template (damaged by a particular dose of ethaRAPTA). Therefore, *A*_d_/*A* is the fraction of non-damaged template at a given dose.

### 3.7. Cell Culture

A human breast adenocarcinoma cell line (MCF-7) and a human colon adenocarcinoma cell line (HT-29) were grown as monolayers in Dulbecco’s modified Eagle medium (DMEM) without phenol red, supplemented with 10% fetal bovine serum and 1% penicillin-streptomycin and incubated at 37 °C with 5% CO_2_.

### 3.8. Cell Treatment and Genomic DNA Preparation

About 1 × 10_6_ MCF-7 or HT-29 cells were seeded into each well of a 6-well flat-bottomed plate. The cells were treated with the RAPTA complexes at various final concentrations (0–400 μM for ethaRAPTA and 0–1000 μM for RAPTA-C), and incubated at 37 °C for 48 h. Genomic DNA was prepared using a procedure modified from the literature methods [33,58]. The RAPTA-treated cells and untreated control cells were harvested and washed twice with 0.5 mL of phosphate-buffered saline (PBS). The cells were centrifugation at 3000*g* for 5 min and the pellet resuspended in a 1.5 mL microcentrifuge tube with 500 μL of a cell lysis buffer. The cell lysate was digested overnight at 37 °C with 25 μL of 10% sodium dodecyl sulfate and 2 μL of a proteinase K solution. After digestion was completed, 140 μL of saturated NaCl (6 M) was added and shaken vigorously for 15 s, followed by centrifugation at 3000*g* for 20 min. The protein sediment was left at the bottom of the tube and the supernatant containing the DNA was transferred to another 1.5 mL microcentrifuge tube. DNA was precipitated by addition of 2 volumes of absolute ethanol and centrifuged at 13,000*g* at 4 °C for 20 min. The DNA pellet was washed twice with 70% ethanol, dried and resuspended in 100 μL of double distilled water.

### 3.9. Quantification of DNA Lesions in Cancer Cells

Exon 11 of the *BRCA1* gene (3426 bp) is a model DNA target for studying the effect of the RAPTA complexes (ethaRAPTA and RAPTA-C) on DNA amplification. The total volume of the PCR mixture was 50 μL containing 400 ng of ruthenated genomic DNA template, 0.5 μM of each forward and reverse primer, 300 μM of each dNTP, 2 units of Phusion Hot Start DNA polymerase, 1.5 mM MgCl_2_, 1xPhusion™ GC Buffer. The PCR conditions used for amplifying the RAPTA adduction were as follows: 3 min at 98 °C; 30 cycles of 30 s at 60 °C, 2 min at 72 °C and a final extension for 7 min at 72 °C. PCR products were electrophoresed on 1% agarose gel at 100 V and stained with ethidium bromide. The resulting PCR products were visualized and quantitated using the QPCR analysis.

### 3.10. Cytotoxicity Assays

MCF-7 and HT-29 cells were cultured in Dulbecco’s modified Eagle medium (DMEM) (pH 7.2–7.4) without phenol red supplement with 10% FBS and 1% penicillin-streptomycin. All cells were incubated at 37 °C with 5% CO_2_. The logarithmically growing cells were detached using 0.25 g/L trypsin-ethylenediamminetetraacetic acid (EDTA) solution (Gibco, Birmingham, MI, USA), washed twice with supplemented medium and centrifuged at 1500*g* for 5 min at 25 °C. Cells were plated at density of 5 × 10^4^ cells per well into flat-bottmomed 96-well microplates and cultured in incubator overnight at 37 °C. The ruthenium(II) complex (ethaRAPTA or RAPTA-C) with various concentrations was added in culture medium, and further incubated for 48 h. The medium was removed and washed twice with 100 μL of phosphate buffer saline (PBS). Then, the 3-(4,5-dimethylthiazol-2-yl)-2,5-diphenyl tetrazolium bromide (MTT) solution (final concentration of 0.5 mg/mL) was added to each well and incubated for 3–4 h. The MTT solution was gently removed and the formazan crystals were dissolved with 200 μL of dimethylsulfoxide solution (DMSO). Absorbance was measured at 570 nm using and automated microplate reader. The cell viability rate was calculated as follows: cell viability (%) = (absorbance of the treated wells)/(absorbance of the control wells) × 100%.

## 4. Conclusions

The present study described a detailed investigation of the interaction of ethaRAPTA with the human *BRCA1* gene fragment. We have characterized the ethaRAPTA interaction with DNA, including probing the sequence specific modified DNA structural stability and DNA amplification using the breast cancer suppressor gene 1 (*BRCA1*) of human breast and colon adenocarcinoma cell lines as models. The preference of ethaRAPTA base binding is in the order A > G > T > C. Once modified, the ethaRAPTA-induced *BRCA1* structure has higher thermal stability than the related compound, RAPTA-C, modified equivalents. EthaRAPTA exhibits a higher efficiency than RAPTA-C in inhibiting *BRCA1* amplification. With respect to both compounds, the inhibition of *BRCA1* amplification is more effective in an isolated system than in cell lines. These data provide evidence that will help to understand the process of elucidating the pathways involved in the response induced by ethaRAPTA. In addition, approaching such a gene as a potential molecular target for the novel antitumor ethaRAPTA could be of interest in cancer therapy.

## Figures and Tables

**Table 1:** Inhibition of cancer cell growth and DNA amplification by ethaRAPTA and RAPTA-C.

Metal complexes	50% Inhibition (μM) of DNA amplification		50% Inhibition (μM) of cancer cell growth	

	MCF-7	HT-29	MCF-7	HT-29
Cisplatin	25 [33]	ND	36	17
ethaRAPTA	300	>300 *	20	50
RAPTA-C	1000	500 *	>1600	500

* ethaRAPTA above 300 μM and RAPTA-C above 600 μM (as shown in Figure 10B) precipitated in the growth medium of the cell culture.

ND = not determined.
